# Clinical benefit of methotrexate plus vinorelbine chemotherapy for desmoid fibromatosis (DF) and correlation of treatment response with MRI

**DOI:** 10.1002/cam4.2374

**Published:** 2019-07-13

**Authors:** Katrina M. Ingley, Sally M. Burtenshaw, Nicole C. Theobalds, Lawrence M. White, Martin E. Blackstein, Rebecca A. Gladdy, Seng Thipphavong, Abha A. Gupta

**Affiliations:** ^1^ Department of Medical Oncology and Hematology Princess Margaret Cancer Centre Toronto ON; ^2^ Division of Medical Oncology and Hematology Sinai Health System Toronto ON; ^3^ Division of General Surgery Sinai Health System, Department of Surgery, University of Toronto Toronto ON; ^4^ Department of Surgical Oncology, Princess Margaret Cancer Centre, Department of Surgery University of Toronto Toronto ON; ^5^ Toronto Joint Department of Medical Imaging University Health Network, Sinai Health System and Women's College Hospital Toronto ON; ^6^ Department of Medical Imaging University of Toronto Toronto ON

**Keywords:** aggressive fibromatosis, desmoid fibromatosis, methotrexate/vinorelbine chemotherapy, MR imaging, T2 signal, tumor volume

## Abstract

**Background:**

Desmoid fibromatosis (DF) is a rare fibroblastic proliferation that was historically treated with surgery. We report (a) outcomes using low‐dose chemotherapy, methotrexate (MTX), and vinorelbine (VNL) for patients with progressing disease (PD) and (b) whether tumor volume (*V*_tumor_) and T2 signal on magnetic resonance imaging (MRI) are more reflective of treatment response compared with maximum tumor dimension (*D*_max_) defined by RECIST1.1.

**Methods:**

Patients with biopsy‐proven DF, treated with MTX/VNL from 1997 to 2015 were reviewed. MRI for a subset of patients was independently re‐evaluated for response by RECIST, *V*_tumor_, and quantitative T2 hyperintensity.

**Results:**

Among 48 patients treated for a median 19 months MTX/VNL, only nine (19%) had previous surgery. RECIST‐based overall response rate was complete response (CR) 20 (42%) + partial response (PR) 19 (39%), stable disease (SD) 8 (17%), for a clinical benefit rate of 98%. The median progression‐free survival (PFS) was 120 months, (95%CI 84‐155 months). Thirty‐six (75%) patients had not progressed at a median 38 months from treatment completion. Most common grade 1/2 toxicities included nausea (n = 12, 25%) and fatigue (n = 9,19%) with no grade 3/4 toxicities. In 22 patients with serial MRIs, there was a decrease in *D*_max_ mean by 30%, *V*_tumor_ by 76%, and in 19/22 (86%) a decrease in T2 signal intensity.

**Conclusion:**

Low‐dose MTX/VNL for a defined duration has high efficacy with sustained benefit and minimal toxicity for treating DF. *V*_tumor_ and T2 signal might better predict treatment response than RECIST.

## 1 INTRODUCTION

Desmoid fibromatosis (DF) is a rare, benign but locally aggressive, and infiltrative fibroblastic tumor that lacks the ability to metastasize but often requires intervention due to pain and functional impairment. The incidence of DF is 0.2‐0.5 per 100 000 individuals per year.^1^ Management of DF has evolved to eliminate unnecessary morbidity from surgery and radiation, toward first‐line active surveillance.^2^, ^3^, ^4^, ^5^, ^6^ An initial watchful waiting approach has not been shown to compromise outcomes when compared with upfront systemic treatment.^6^, ^7^

When there is persistent progression of DF, many expert centers currently advocate for the use of medical therapy for initial intervention.^8^, ^9^, ^10^ Although there are several systemic therapeutic DF options, in the absence of comparative studies, most institutions have selected a regimen based on historical data and local experience. Furthermore, selection of systemic treatment is individualized due to the variable natural history of DF and patient factors, such as pregnancy planning, fertility, and quality of life. The preferred DF regimen at the University of Toronto has been systemic low‐dose chemotherapy with methotrexate plus vinorelbine (MTX/VNL) based on response rates, ease of administration, and patient tolerability. Other expert centers have also reported their experience with MTX and vinca alkaloid regimens and a range of best response rates, from 15% to 52%,^1^, ^8^, ^11^, ^12^, ^13^, ^14^ has been observed in mostly pretreated, heterogenous DF patient populations. Previous literature has described using low‐dose chemotherapy for DF in heavily pretreated cohorts, in whom prior surgery 53%‐80% and radiation 10%‐17% were used.^8^, ^11^, ^14^ Our study reports best response in a population minimally exposed to these prior interventions.

The assessment of response to medical therapies using traditional RECIST‐based evaluation is limited in DF as complete tumor regression is not necessary for a successful clinical outcome. DF configuration, especially those occurring in the abdominal wall, is elongated or ellipsoid in configuration and a single measurement (*D*_max_) may not be sensitive for clinical treatment response.^15^ The use of magnetic resonance imaging (MRI) can be beneficial in assessing response to therapy in DF.^15^, ^16^ Specifically, decrease in T2‐weighted imaging lesional signal intensity reflects treatment response, and this correlates with loss of cellularity and higher collagen on pathological DF examination.^15^, ^17^ Treatment response by RECIST criteria may not be adequately described by the changes in largest dimension of these tumors (*D*_max_); we evaluated the approximate change in tumor volume (*V*_tumor_) to assess if this method of measurement could compliment T2 imaging changes in evaluation of treatment response.

Thus, we report one of the largest studies to date on the efficacy and toxicity of MTX/VNL in an adult DF population treated uniformly at a single institution and in a subset of patients, we report T2‐weighted signal changes based on review of serial MRI images.

## 2 METHODS

### 2.1 Patient characteristics

Patients with biopsy‐proven DF at Mount Sinai Hospital/ Princess Margaret Cancer Centre from 1997 to 2015 were identified from a prospective database. A retrospective chart review was performed on 48 patients who received MTX/VNL chemotherapy for progressing DF. Data collected included demographics, treatment details, and toxicity. The study was reviewed and approved by the institutional research ethics boards at Mount Sinai Hospital and Princess Margaret Cancer Centre.

### 2.2 Treatment

The therapy included MTX 25 mg/m^2^ plus VNL 25 mg/m^2^ intravenously on days 1, 8, and 15 every 28 days for a planned maximum duration of 24 cycles. Reason for early discontinuation of treatment was recorded. Toxicity to chemotherapy treatment was graded using the CTCAE version 4.0. Chemotherapy was held if absolute neutrophil count was <1.5 × 10^6^. Chemotherapy doses missed were omitted and not compensated for at a later time point.

### 2.3 Radiological Treatment Response

A dedicated sarcoma radiologist (ST) reviewed a subset of 22 patients with serial MRI scans to evaluate radiologic treatment response by RECIST, volume, and T2 signal changes, at pretreatment, 3‐6 months following start of treatment and within 9 months of completing treatment, with rare exceptions outside these time frames. MRI examinations were performed on 21 of 22 patients utilizing dedicated institutional abdomen, pelvic, or musculoskeletal protocols dependent on the site of disease (1.5T Aera, 1.5T Avanto Fit, 3T Skyra, 3T Skyra Fit, Siemens); one patient had MR imaging performed at an outside center. Largest dimension on imaging (*D*_max_), *V*_tumor_, and semiquantitative T2 hyperintensity classification using interquartile range scoring on MRI were compared. On T2‐weighted or T2‐weighted fat‐saturated MRI images, tumors were ranked as containing: 0%‐25%, 25%‐50%, 50%‐75%, or 75%‐100% of internal high T2 signal intensity. High T2 signal intensity was defined as signal comparable to fluid. *V*_tumor_ was approximated using an elliptical volume equation (*V* = *π*/6*L*W*H). Radiologic treatment response was defined as “decreased” if T2 signal quartile decreased from baseline.

All patients within the cohort were assessed by standard RECIST version 1.1 criteria using combination imaging with ultrasound, CT, and MRI and were not fully evaluated by MRI at every time point. Response was evaluated at the end of therapy and point of last contact. Best overall response is defined as the best response across all time points.^18^

### 2.4 Statistical analysis

Standard descriptive statistics were calculated for continuous variables (median, range) and categorical variables (number and percentage) to characterize the patient demographics, response, and toxicity experience. A univariate analysis was performed for all continuous data with the median (range) reported. T2 changes evaluated on serial MRI images from the subgroup of 22 patients were compared at interquartile ranges. Progression‐free survival (PFS) was calculated as the time (months) from the first day of chemotherapy treatment to the last known follow‐up, progression/recurrence, or death. PFS was estimated using Kaplan‐Meier analysis and significance determined using log‐rank analysis. Standard errors (%) are reported for PFS. All statistical analyses were performed using SPSS ver. 25.

## 3 RESULTS

### 3.1 Patient characteristics

Patient characteristics are summarized in Table 1. The median patient age was 33 years (range, 13‐73) and 31 (65%) of patients were female. Tumors were located in the extremity 16 (33%), abdominal wall 13 (27%), head and neck 4 (8%), trunk 6 (13%), mesentery 7 (15%), and 2 (4%) were multifocal. The median greatest tumor dimension was 9.4 cm (range 3.2‐19.2 cm). The majority of 37/48 (77%) patients had primary disease and went on to progress within our institution during observation, hormonal, and/or NSAID therapy. The remaining 11 (23%) of patients were treated at outside hospitals where initial management decisions were made and referred to our institution for management of disease recurrence or progression.

**Table 1:** Patient characteristics, prior treatment modalities

Total number	N = 48
Gender	N (%)
Female	31 (65)
Male	17 (35)
Age: median [range]	33 [13‐73]
Location of DF	N (%)
Extremity	16 (33)
Abdominal wall	13 (27)
Head & neck	4 (8)
Trunk	6 (13)
Mesentery	7 (15)
Multifocal^a^	2 (4)
No prior therapy	19 (40)
Prior therapy	29 (60)
Surgery alone	5 (17)
Tamoxifen alone	15 (53)
NSAIDS alone	2 (7)
Surgery and NSAIDS	1 (3)
Surgery and Tamoxifen	2 (7)
Tamoxifen and NSAIDS	2 (7)
Surgery, radiation and NSAIDS	1 (3)
Multiple drug treatments (including doxorubicin)	1 (3)

a Multifocal tumors were located in the abdominal wall and mesentery, and in association with FAP.

Twenty‐nine (60%) patients received previous treatment, of which 19 (39%) received medical therapy only, either at our institution or at another hospital prior to starting low‐dose chemotherapy. There were 19 (40%) patients who were entirely treatment naive during a period of observation prior to MTX/VNL (Table 1). The majority, 46 (96%), had no prior exposure to doxorubicin or radiation and only nine (19%) patients (all pretreated at an outside hospital) had received previous surgery alone or part of multimodality therapy (Table 1). Four patients within the entire cohort had familial adenomatous polyposis (FAP).

### 3.2 Treatment and toxicity

**Figure 1:**
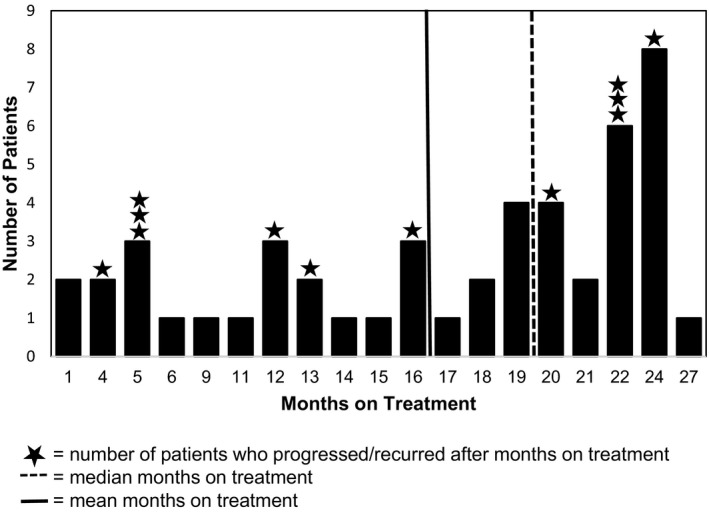
Duration of therapy for all patients and for those in whom disease progressed. Duration of therapy, in months, for all patients and duration of therapy received by those patients who progressed at some point in their treatment or follow‐up. Star‐ indicates a patient who has progressed and their total duration of treatment received

The median number of months on treatment was 19 (range 1‐27) (Figure 1). Forty‐four (92%) patients received their scheduled chemotherapy doses; three (6%) were given the same dosing on day 1 and 8 every 21 days for the same planned maximum duration. One patient had a complex dose reduction schedule over a shortened period of treatment, 6 months. The majority of patients, 27 (56%), completed 18 or more cycles of therapy; nine (19%) completed the full 24. Thirty‐eight (79%) patients discontinued chemotherapy prior to 24 months for the following reasons: intolerance (fatigue and alopecia), 2 (4%); response achieved (ultimately determined as per physician discretion), 28 (58%); patient preference, 8 (17%). One patient discontinued treatment due to progressive disease, 1 (2%).

The most common grade 1/2 toxicities were nausea, reported in 12 patients (25%), fatigue in 9 (19%), or concurrent fatigue and nausea in 4 (8%). Neutropenia grade 1/2 was also observed in four (8%) of patients. Other reported grade 1/2 toxicities have been summarized in Supplemental Table 1. There were no grade 3/4 toxicities observed. Schedule modification occurred in four (8%) patients, due to nausea alone, (n = 2), nausea and anemia, (n = 1), and abdominal cramping, (n = 1).

### 3.3 RECIST response at end of treatment

At the end of therapy, RECIST‐based response was as follows: complete response (CR) 20 (42%), partial response (PR) 19 (39%), stable disease (SD) 8 (17%), and progressive disease (PD) 1 (2%), respectively, for a clinical benefit rate (CR + PR+SD) of 98%. One patient, age 62 years, who progressed after 5 cycles, presented with a chest wall DF that recurred after surgery and was subsequently re‐excised without disease recurrence.

### 3.4 Progression‐free survival

**Figure 2:**
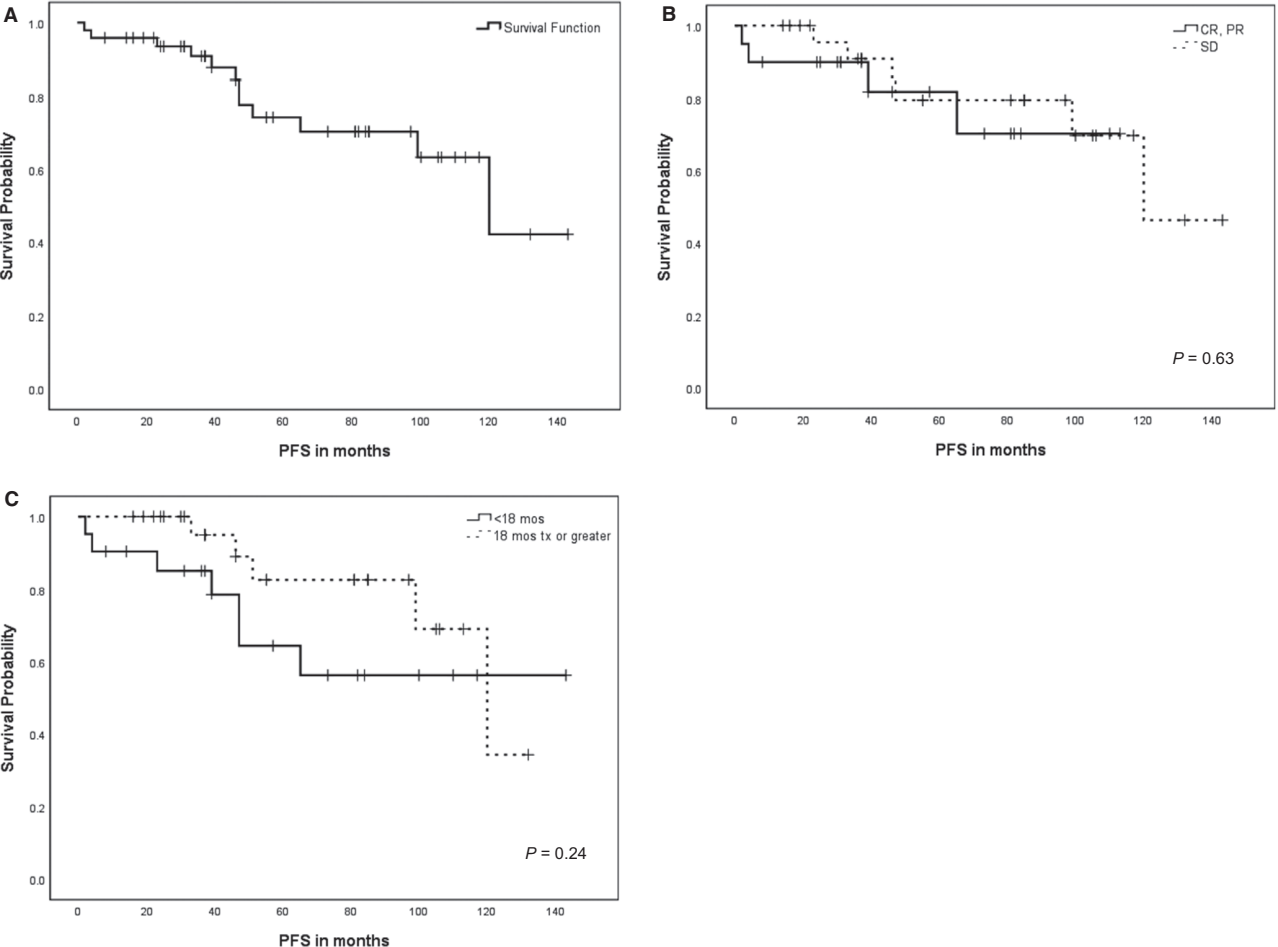
Kaplan‐Meier progression‐free survival curves. Kaplan‐Meier progression‐free survival (PFS) for: A. Entire cohort of 48 patients, B. Clinical benefit rate, complete response (CR) + partial response (PR) versus stable disease (SD), *P* = 0.63, C. Treatment length, <18 months vs ≥ 18 months of treatment, *P* = 0.24

The median PFS for the overall population (n = 48) was 120 months (range 2‐143 months, 95%CI 84‐155 months) with a 5‐year PFS of 74.4 ± 7.6% (Figure 2A). There was no significant difference in 5‐year PFS between those patients who obtained CR/ PR, 81.8 ± 10% compared to those that achieved SD, (79.5 ± 9.2%, *P* = 0.63) (Figure 2B). PFS was evaluated based on treatment length ≥ 18 months (n = 27) and <18 months (n = 21); 5‐year PFS was 82.7 ± 9.2% and 64.3 ± 12.1%, respectively (*P* = 0.24) (Figure 2C).

### 3.5 Durability of response

**Figure 3:**
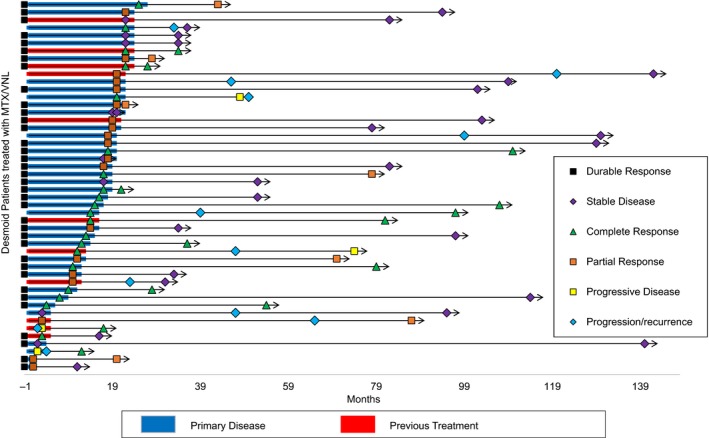
Treatment duration, follow‐up, and response for all patients. Swimmers plot demonstrating the individual patients' treatment duration, follow‐up, and response at end of treatment and point of last follow‐up. Each bar represents one subject in the study. Treatment duration is shown by primary presentation (refers to patients within our institution in which disease progressed, PD, during observation, hormonal, or NSAID therapy) versus recurrent/residual presentation (treated at outside hospitals and referred for PD) from time zero, that is, from start of MTX/VNL treatment (blue and red horizontal bars, respectively). Response at treatment end is highlighted (refer figure key). Durable response is defined as a subject who has confirmed response‐ CR, PR, or SD at last follow‐up without recurrence. Length of follow‐up (solid arrows) and response at last point of contact is documented

Thirty‐six patients (75%) remained free from disease progression without the need for new treatment at a median follow‐up from the end of MTX/VNL of 38 months (range 1‐139). A total of 12 patients progressed after discontinuation of MTX/VNL (median time to progression, 26 months after completing treatment, range 5‐95 months) having obtained at least a PR (n = 9) (Figure 3). Therapy was stopped prior to 18 months in four of these nine patients due to response; physician preference (n = 3) (ranging between 5 and 13 months) and patient preference (n = 1) at 16 months.

Of the 12 patients (25%) who developed progression, nine patients had subsequent intervention: surgery (n = 2), surgery plus radiation (n = 2), sorafenib (n = 4) and two patients were re‐challenged with MTX/VNL; both were stable at last follow‐up (Table 2). For the other 3/12 patients who progressed after chemotherapy, one died of FAP complications; one remained asymptomatic and stable off any treatment, and one patient is considering clinical trials for PD. One patient had PD 2 years following completion of therapy in the context of pregnancy. There were no patients with isolated abdominal wall DF that progressed on low‐dose chemotherapy.

**Table 2:** Second‐line therapies, clinical outcomes, and response for 12 patients who progressed on MTX/VNL

Patients with a first event (progression, recurrence, death)	Age (yr) at diagnosis	Greatest dimension of initial DF (cm) at start MTX/VNL	Site	Duration MTX/VNL (mo)	PFS (mo)	Clinical Details
1	32	12.2	Trunk	5	47	Prior tx: none. Patient decided to stop MTX/VNL due to nausea. Received RT and surgery; recurred at 4 y, when son was 6 mo age. No further tx. Remains with SD
2	19	5.2	Abdo wall + Mesenteric‐ multifocal	5	65	Prior tx: surgery + tamoxifen. Died of FAP‐related complications at 7 y. SD at LFU
3	30	7.4	Extremity	16	39	Prior tx: none. PD during pregnancy. NSAID's, Tamoxifen, Sorafenib for 18 mo with CR. SD at LFU
4	45	7.4	Trunk	24	33	Prior tx: none. Recurrence at 1 y post MTX/VNL; slow PD over 2 y. No further Tx. SD at LFU
5	54	6.6	Trunk	22	46	Prior tx: none. 1st recurrence: RT + surgery, 2nd recurrence 3 y after surgery: re‐challenge with MTX/VNL chemo × 18 mo. SD at LFU.
6	33	13.4	Extremity	22	120	Prior tx: surgery. Recurrence treated with Sorafenib × 12 mo, stopped due to toxicity. SD at LFU
7	28	9.2	Head & Neck	20	99	Prior tx: tamoxifen. Sorafenib × 12 mo for progression, stopped due to toxicity. SD at LFU
8	29	14.6	Head & Neck	12	23	Prior tx: multiple medical therapies including cytotoxic chemotherapy, hormonal therapy and TKIs. Progressed and rechallenged with MTX/VNL for 6 mo. SD at LFU
9	62	11.3	Trunk	5	2	Prior tx: surgery. PD on MTX/VNL treated with surgical resection, with CR
10	30	10.9	Abdominal wall‐ multifocal	22	51	Prior tx: none. Persistent slow PD (doubled long axis tumor over 1.5 y); patient deciding on treatment, currently asymptomatic
11	32	7.5	Extremity	13	47	Prior tx: surgery + RT +NSAIDs; now on Sorafenib
12	45	6.7	Mesenteric	4	4	Prior tx: none. Minimal PD plus intolerant to chemo; moved to surgery

Abbreviations: Durations: mo, month; y, year. Treatment (tx): MTX/VNL, methotrexate and vinorelbine; PD, progressive disease; LFU, last follow‐up; RT, radiation. Response: CR, complete response; SD, stable disease; TKI, tyrosine kinase inhibitor.

### 3.6 RECIST response at last contact

At last point of contact, (median 56 months, range 14‐145), 46/48 (96%) patients were alive and two patients (4%) still had active disease. The two deceased patients both had PR to MTX/VNL and died of other causes: one due to complications of FAP and the other from esophageal cancer.

### 3.7 MRI imaging to assess response to treatment

#### 3.7.1 *D*_max_ and *V*_tumor_

Serial MRIs were available in 22 patients to assess response to therapy, by traditional RECIST criteria (using *D*_max_), *V*_tumor_, and T2 signal changes. For these 22 patients, DF site was extremity 9(41%), abdominal wall 7(32%), head and neck 3(14%), trunk 2(9%), and mesentery 1(4%) similar in proportion to the overall cohort. At end of treatment, (median 20 months, range 9‐27), the mean *D*_max_ decreased by 30% and *V*_tumor_ decreased by 76%, showing greater sensitivity in treatment response using a volumetric approach to DF measurement.

#### 3.7.2 T2 response

**Figure 4:**
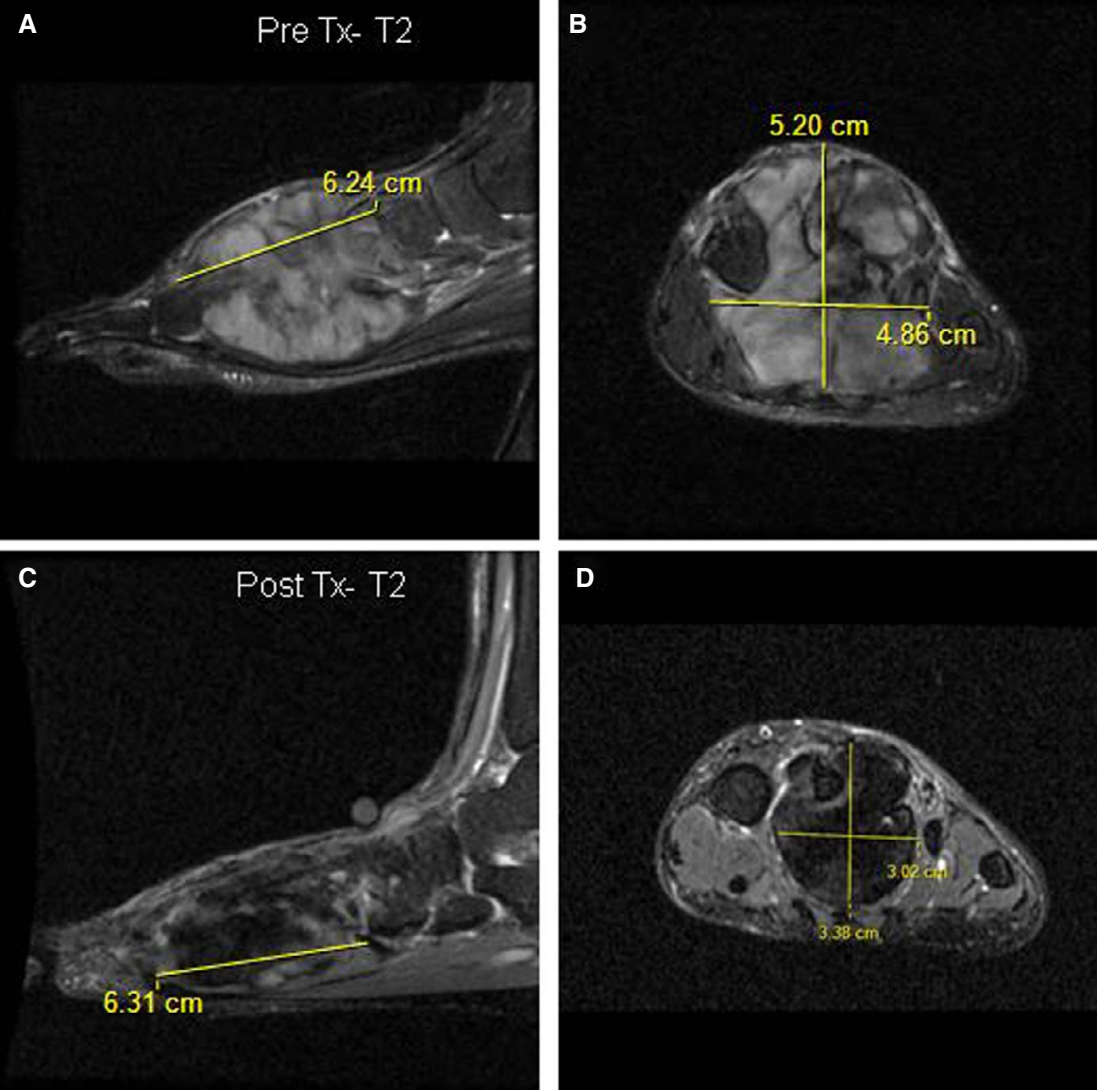
MRI imaging showing T2 characteristics of DF in foot in response to MTX/VNL. T2‐fat‐saturated sagittal and coronal MRI images of DF in left foot pre‐ (A and B) and post‐ (C and D) MTX/VNL. No response to treatment (Tx) as per *D*_max_ with no change in the longest axis of the DF (6.2 vs 6.3 cm), compared with significant treatment response seen by decreased *V*_tumor_ (83 to 34 cm^3^) and T2 intensity (50%‐75% to 0%‐25%)

Qualitative change in T2 signal intensity was evaluated at the pre‐ and posttreatment MRI. In almost all patients, 21 (95%), high T2 signal intensity involving 50% to 100% of the lesion was observed pretreatment. By the end of treatment, 18/22 (82%) of patients had an internal T2 signal intensity that ranged between 0% and 50% (Table S2) and 19/22 (86%) had developed a decrease in T2 signal intensity. At last follow‐up, 16/22 (73%) had a sustained response (CR n = 6, PR n = 3, SD n = 7 by RECIST) at a median of 25 months (range 1‐109) from end of treatment. This illustrates that a measurable response to treatment can be demonstrated by a decrease in *V*_tumor_ and T2 intensity despite no change in *D*_max_ (Figure 4).

## 4 DISCUSSION

In adults with DF, treatment with low‐dose MTX/VNL chemotherapy, for a maximum of 24 months, is well tolerated and effective with sustained benefit. In our series of 48 patients treated with low‐dose MTX/VNL for documented progressive disease, 81% of patients achieved CR/ PR by RECIST 1.1 criteria as best overall response with 98% clinical benefit rate comparing favorably with other studies trialing similar regimens^8^, ^13^ (Table 3). The median PFS of 120 months (95%CI 84‐155 months) for our cohort demonstrates that response is achieved during treatment and clinical benefit is sustained throughout the follow‐up period for the majority of patients. These outcomes support the median PFS of 75 months for the overall cohort of a large study (n = 75) treated on combinations of MTX and vinca alkaloids treated for median duration of 14 months.^8^ Durability of benefit upon completion of therapy was also apparent in our study; 75% of patients had disease stability at a median of 38 months from end of chemotherapy. Furthermore, two patients responded favorably to re‐challenge with low‐dose chemotherapy after recurrence, supporting previous reports that treatment can be repeated with benefit.^14^

**Table 3:** Comparison of efficacy between intravenous regimes that incorporate MTX and vinca alkaloids for DF, that is recurrent/ progressive/ advanced/ or not amenable to surgery/radiation, by treatment duration

Study	Number (n)	Age(yr)	Treatment	Duration of treatment (median)	Prior treatment number (%)				Overall response rate (%)(CR + PR)	Clinical benefit (%)(CR + PR+SD)
					S	R	M	C		
Garbay et al^11^	62	30	Variable regimens MTX/VBL 27 (43.5)	18 wk	33 (53)	8(13)	37(54.7)	—	21(MTX/VBL‐ 15)	80.6
Skapek et al^12^	26	10	MTX/VBL	40 wk	—	—	——	—	19.2	69.2
Li et al^13^	71	14	MTX/VNL	12 mo	—	—	——	—	35.2	87.3
Azzarelli et al^14^	30	27	MTX/VBL	12 mo	24 (80)	5(17)	4(13)	3(10)	40	100
Palassini et al^8^	75	36.6	Variable Regimens MTX/VNL43 (57.3)	14 mo	54(72)	8(10)	36(48)	—	48 (MTX/VNL‐ 51.2)	98.7
Park et al^1^	22	32	MTX/VBL	12 mo	14(64)	—	4(18)	2(9)	52	95
Ingley et al	48	33	MTX/VNL	19 mo	9(19)	1(2)	23(48)	1(2)	81	98

**Abbreviations: **n = Number of patients evaluated for response. Age‐ median age of diagnosis/ onset, in years.

Treatment: MTX, methotrexate; VNL, vinorelbine; VBL, vinblastine; n(%) of patients reported who were administered MTX/ vinca alkaloid.

Duration of treatment: when median duration of treatment was not provided, anticipated treatment course was documented; wk, weeks; mo, months.

Prior treatment: S, surgery; R, radiation; M, at least one or more medical treatments‐ NSAIDs, antiestrogens, tyrosine kinase inhibitors; C, chemotherapy.

Clinical benefit using low‐dose chemotherapy for DF has been supported by other reports that included children and adolescents, including a Phase II prospective study that avoided potential long‐term morbidity from surgery and radiation.^12^, ^13^ Outcomes from the current study compare favorably to the clinical benefit rate/ 5‐year PFS of 87%/ 36.3% reported in a retrospective series of 71 patients (53% <18 years age) who were not surgical candidates and treated with low‐dose chemotherapy for 1 year.^13^ The relative treatment naivety of our population, longer treatment duration, and standard first‐line chemotherapy approach established within a multidisciplinary setting could contribute to our favorable response rates.

The length of systemic treatment of DF is a challenge for patients, as this susceptible population tends to be dominated by young females in childbearing years. Since it is crucial that women avoid pregnancy due to chemotherapy teratogenicity, the need to postpone childbearing until treatment completion can influence decision‐making of treatment duration. Optimal duration of systemic treatment for DF has varied in the literature but PFS favors longer duration of treatment for at least 1 year.^8^, ^12^, ^14^ Continuing low‐dose chemotherapy, despite early CR/PR, for closer to 2 years (if no significant toxicity) may facilitate a more durable response (Table 3). In contrast to other medical therapies offered in DF, which have no fixed duration, a defined prescription of time on therapy can be helpful for life planning.

Treatment with low‐dose MTX/VNL is well tolerated with minimal toxicity. Prior reports of MTX‐based treatment suggest excessive toxicity^14^, ^19^—however, this may have been due to inclusion of heavily pretreated patients, higher doses, and shorter intervals between cycles. In our study, there were no grade 3/4 toxicities observed. In comparison, the use of vinblastine does not appear to be as well tolerated compared to VNL, with higher toxicity rates, especially neurotoxicity, hepatotoxicity, and neutropenia; however, this may also reflect higher doses and frequent scheduling.^1^, ^10^, ^12^, ^14^, ^19^

**Figure 5:**
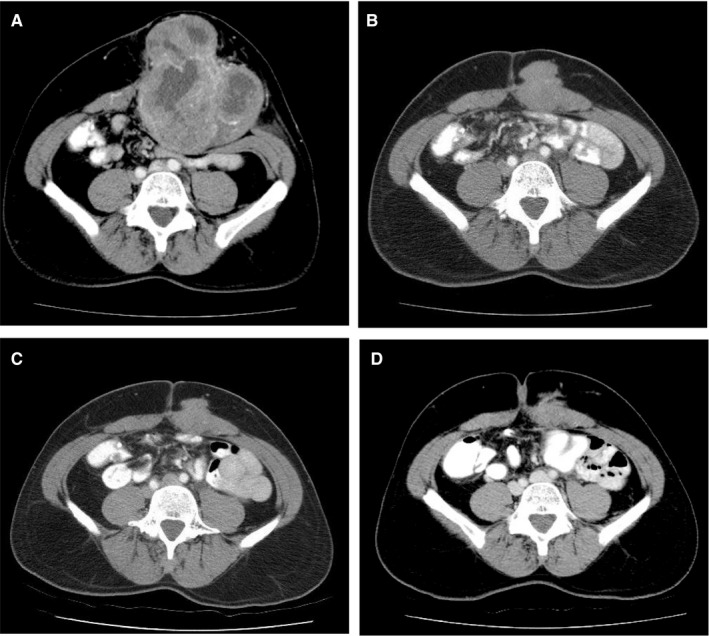
Sustained response of DF to MTX/VNL on MRI imaging. Response of DF on MRI throughout treatment with MTX and VNL, showing continued response despite treatment stopping after 25 cycles. A. Pretreatment, B. After 12 cycles, C. Posttreatment, 1 month after treatment end, D. Posttreatment, 2 months after treatment end

Evaluating response to treatment of DF radiologically is in evolution. In this study, independent radiology review demonstrated that treatment response of DF to MTX/VNL correlated highly with *V*_tumor_ and T2 intensity evaluated by MRI, findings also supported by Sheth.^15^ Characteristic MRI findings anticipated with tumor response include shrinking and/or reduced T2 signal intensity (Figure 5). The maximum tumor dimension or *D*_max_ remained relatively constant, whereas decreased *V*_tumor_ reflected response to MTX/VNL in half of the study participants. Monitoring change in *V*_tumor_ and T2 signal may be more sensitive in detecting treatment response than RECIST criteria allowing patients to continue a full chemotherapy schedule to achieve a maximal clinical benefit. Braschi‐Amirfarzan et al^20^ presented revised criteria for response assessment that incorporates “modified response” (mR) and “modified progression” (mP) that rely on MRI input when tumor size could be unchanged. We propose the integration of a similar modified response for MRI evaluation of DF especially since limiting the use of repeated radiation doses using other imaging modalities such as CT is desirable in young healthy patients.

The molecular basis of DF is known to be due to disruption of the Wnt pathway as germline inactivating mutations in the adenomatous polyposis coli (*APC*) gene which is responsible for familial DF, whereas sporadic DF often harbor activating mutations (T41A and S45F) in exon 3 of the *β*‐catenin encoding gene *CTNNB1*, that causes accumulation of *β*‐catenin.^21^ Although mutational status may provide prognostic information with increased local recurrence risk associated with *β*‐catenin S45F mutation compared to wild‐type DF,^22^, ^23^, ^24^ currently our ability to prognosticate patient outcome and select aggressive biology that requires intervention is limited. There has been recent interest in other medical therapies including NOTCH inhibitors,^25^, ^26^ tyrosine kinase inhibitors—imatinib,^27^, ^28^ nilotinib,^28^ sorafenib,^29^, ^30^ and pazopanib.^31^, ^32^ These therapies are advantageous in that delivery is oral, negating the need for multiple hospital visits. Tolerance to some of the agents may limit dosing, which was originally defined within cancer populations. As with many other molecular therapies, the total duration of treatment also remains unknown. Future studies investigating systemic treatment for DF will likely include mutation status analysis that may help to guide treatment decisions.

Although retrospective, DF patients were managed at an expert single center with uniform multidisciplinary input, including surgical and medical oncology, pathology, and radiology. Analyses from one dedicated sarcoma radiologist with expertize in abdominal radiology were used for all blinded single reads. Potentially, this could have contributed to study bias. Treatment decisions were made with careful review based on tumor response and patient factors. Evaluating patient symptomatic response was outside the scope of this study but it would be ideal in future prospective studies to correlate radiological findings with symptomatic benefit.

## 5 CONCLUSION

This is one of the largest series of adults treated uniformly with MTX/VNL for their DF demonstrating clinical benefit. Its low toxicity profile, defined treatment length and lack of long‐term toxicity supports its use as a reasonable choice for patients and providers. Our data suggest that offering MTX/VNL as first‐line with a defined maximum duration of treatment of 24 months may be worthy of consideration, especially when oral agents are unavailable due to cost. Furthermore, the defined length of treatment prescription with durability of response is favorable. Monitoring medical treatment with an estimated *V*_tumor_ and degree of T2 signal intensity change may be a better predictor of response than *D*_max_.

## FUNDING SUPPORT

No specific funding was disclosed.

## CONFLICT OF INTEREST

The authors declare no conflict of interest.

## AUTHOR CONTRIBUTION

KI participated in data interpretation, helped create the tables and prepared the manuscript with revisions based on coauthor's feedback. SB participated in data curation and formal analysis, created the data figures and manuscript preparation. NT participated in data curation and extraction. LW performed radiological analysis. MB was responsible for study conception and design, methodology and oversaw data collection. RG participated in study conception; manuscript edits and project administration. ST provided imaging, performed radiological analysis and participated in manuscript revisions. AG participated in study conception, reviewed and edited the manuscript, provided supervision and oversaw completion of the project.

## Data Availability

The data that support the findings of this study are available from the corresponding author upon reasonable request.
