# Supplementary material for: Clinical benefit of methotrexate plus vinorelbine chemotherapy for desmoid fibromatosis (DF) and correlation of treatment response with MRI
Source: Cancer Med. 2019 Jul 13;8(11):5047–57. doi: 10.1002/cam4.2374 (PMC6718598; doi:10.1002/cam4.2374)
Supplement: Supplementary file 1 [file CAM4-8-5047-s001.doc]

| **Supplemental Table 1. Toxicities** | | |
| --- | --- | --- |
|  | **Grade 1**  **N (%)** | **Grade 2**  **N (%)** |
| **Total Number** | **33 (66)** | **17 (34)** |
| Nausea | 9 (18) | 7 (14) |
| Fatigue | 8 (16) | 5 (10) |
| Headache | 1 (2) | _ |
| Neutropenia | 3 (6) | 1 (2) |
| Oral dysesthesia | 4 (8) | _ |
| Fever | 3 (6) | _ |
| Weight gain | 1 (2) | _ |
| Pain | _ | 2 (4) |
| Diarrhea | 2 (4) | 1 (2) |
| Anemia | _ | 1 (2) |
| Constipation | 1 (2) | _ |
| Alopecia | 1 (2) | _ |
