# Supplementary material for: Clinical benefit of methotrexate plus vinorelbine chemotherapy for desmoid fibromatosis (DF) and correlation of treatment response with MRI
Source: Cancer Med. 2019 Jul 13;8(11):5047–57. doi: 10.1002/cam4.2374 (PMC6718598; doi:10.1002/cam4.2374)
Supplement: Supplementary file 2 [file CAM4-8-5047-s002.doc]

**Supplemental Table 2**

Change in Dmax, Vmax and T2 After Chemotherapy

|  | | | | **Dmax** | **Vmax** | **T2** | | |
| --- | --- | --- | --- | --- | --- | --- | --- | --- |
| **Pt No.** | **Age** | **Sex** | **Tumor Site** | **% change** | **% change** | **%Pre** | **%End** | **change** |
| 1 | 30 | F | Extremity | -19 | -62 | 50-75 | 0-25 | ↓ |
| 2 | 54 | F | Head and Neck | -15 | -59 | 75-100 | 25-50 | ↓ |
| 3 | 25 | F | Abdominal wall | -38 | -74 | 50-75 | 0-25 | ↓ |
| 4 | 63 | F | Extremity | -43 | -89 | 75-100 | 50-75 | ↓ |
| 5 | 45 | F | Trunk | -23 | -67 | 75-100 | 0-25 | ↓ |
| 6 | 54 | F | Extremity | -21 | -60 | 75-100 | 0-25 | ↓ |
| 7 | 17 | F | Extremity | -30 | -91 | 50-75 | 50-75 | ↔ |
| 8 | 28 | M | Head and Neck | -47 | -85 | 75-100 | 0-25 | ↓ |
| 9 | 33 | F | Abdominal wall | -21 | -93 | 50-75 | 50-75 | ↔ |
| 10 | 19 | M | Extremity | -15 | -62 | 75-100 | 0-25 | ↓ |
| 11 | 43 | F | Extremity | -35 | -85 | 75-100 | 0-25 | ↓ |
| 12 | 32 | F | Extremity | -15 | -30 | 75-100 | 75-100 | ↔ |
| 13 | 28 | F | Abdominal wall | -26 | -80 | 50-75 | 25-50 | ↓ |
| 14 | 41 | F | Trunk | -6 | -69 | 75-100 | 0-25 | ↓ |
| 15 | 14 | M | Extremity | -42 | -87 | 75-100 | 0-25 | ↓ |
| 16 | 23 | F | Abdominal wall | -13 | -84 | 50-75 | 25-50 | ↓ |
| 17 | 27 | F | Mesentery | -40 | -87 | 50-75 | 0-25 | ↓ |
| 18 | 20 | F | Head and Neck | -52 | -86 | 50-75 | 25-50 | ↓ |
| 19 | 30 | M | Abdominal wall | -51 | -90 | 75-100 | 0-25 | ↓ |
| 20 | 39 | M | Abdominal wall | -39 | -90 | 25-50 | 0-25 | ↓ |
| 21 | 36 | F | Abdominal wall | -27 | -75 | 75-100 | 0-25 | ↓ |
| 22 | 32 | F | Extremity | -44 | -73 | 50-75 | 0-25 | ↓ |

Dmax % change= % change in Dmax from pre to end of treatment.

Vmax % change= % change in Vmax from pre to end of treatment

T2% - defined as the quartile percentage of T2 signal hyperintensity within the lesion pre to end of treatment.
